# Abdominal ectopic pregnancy complicated with a large bowel injury: a case report

**DOI:** 10.1186/s13256-021-02713-9

**Published:** 2021-03-22

**Authors:** Abraham Fessehaye, Biruck Gashawbeza, Mekdes Daba, Muhudin Arusi, Tsega Terefe

**Affiliations:** 1 Department of Obstetrics and Gynecology, Saint Paul’s Hospital Millennium Medical College, Addis Ababa, Ethiopia; 2 St. Paul’s Hospital Millennium Medical College, Addis Ababa, Ethiopia; 3 Department of Surgery, Saint Paul’s Hospital millennium Medical College, Addis Ababa, Ethiopia

**Keywords:** Abdominal ectopic pregnancy, Bowel injury, End-to-end anastomosis

## Abstract

**Background:**

Abdominal pregnancy accounts for 0.6 to 4% of all ectopic pregnancies. Due to delays in diagnosis and difficulties in the management of abdominal pregnancy, the risk of mortality is significantly higher than for uncomplicated ectopic pregnancies.

**Case summary:**

A 23 years-old gravida-II, ectopic-I Ethiopian woman was initially managed as a case of missed second trimester abortion. Later on, abdominal ectopic pregnancy was diagnosed with ultrasound and she underwent a laparotomy. Though fetus and placenta was removed successfully without significant hemorrhage, there was inadvertent sigmoid colon injury.

**Conclusion:**

In the management of abdominal ectopic pregnancy, the possibility of bowel injury during entry to the abdominal cavity at laparotomy should always be considered and an experienced general surgeon should always be in attendance before opening the abdomen, to prevent it from happening.

## Background

Abdominal pregnancy accounts for 0.6 to 4% of all ectopic pregnancies. It is seen 1 in 3372 to 1 in 21,439 live births. Mortality rates are 7.7 times higher than in tubal pregnancy, and 89.8 times higher than in intrauterine pregnancy. Due to delays in diagnosis and difficulties in the management of abdominal pregnancy, the risk of mortality is significantly higher than for uncomplicated ectopic pregnancies.

High index of suspicion is important to diagnose abdominal pregnancy. In contrast to tubal ectopic pregnancies, abdominal pregnancies may go undetected until an advanced gestational age. The mainstay of management is surgery. Removal of the ectopic pregnancy mass could cause intractable hemorrhage and/or organ injury because of deep trophoblastic invasion into the surrounding tissue.

## Case presentation

A 23-year-old Gravida-II, Ectopic-I Ethiopian woman was referred from a district Hospital as a case of failed medication abortion at gestational age of 24 weeks plus 6 days from a reliable date. She presented with a history of vaginal bleeding of 3 days duration. She had no history of abdominal pain, nor fever, nor bowel habit changes, nor vomiting. She also had no history of urinary symptoms. Her past surgical, medical, and family history was unremarkable.

Before referral, she was provided repeated cycles of 5 dose regimen misoprostol 200 mg vaginally with a diagnosis of second trimester missed abortion. She was refereed for possible dilatation and evacuation procedure once the medication abortion failed.

Her first pregnancy was an ectopic tubal pregnancy for which right salpingectomy was done after a ruptured right tubal ectopic pregnancy was diagnosed with pelvic ultrasound on background of classic clinical presentation—amenorrhea, abdominal pain, and vaginal bleeding. The intra-operative findings at that time was a ruptured right ampullary pregnancy with significant hemoperitonium.

Up on her arrival to our Hospital, she was immediately evaluated by a senior Ob-Gyn consultant and a diagnosis of abdominal ectopic pregnancy was made based on ultrasound findings of an empty uterus with a well formed demised fetus in the abdominen with femoral length that corresponded 24 weeks of gestation. With full preparation made for possible need of massive blood transfusion, patient underwent a laparotomy surgery with a generalist Ob-Gyn consultant and a gynecology oncology fellow in attendance.

The intra-operative finding was 10 by 9 cm sized abdominal ectopic pregnancy buried in a dense adhesion, covered with loops of the large bowel that was adherent to it’s surface. The right ovary was invisible due to firm adhesion. The left tube and ovary were healthy looking. There was no any finding suggestive of uteroperitoneal fistula.

Carefully, the mass was dissected from surrounding organs but there was inadvertent serosal sigmoid colon injury. Intra-operative consultation was made to a general surgeon. The whole colon was inspected, injury site identified, and adhesion lysis was done successfully. A 200 g weighing macerated fetus along the placenta was extracted without any difficulty. End-to-end anastomosis was done for the sigmoid colon injury.

Our patient didn’t require any blood transfusion and was discharged after 5 days of post-operative recovery in good condition. She was reevaluated on subsequent follow ups, after a week and 2 weeks respectively. She had no any complaint and her clinical profile didn’t document any abnormality during the two follow-up visits.

## Discussion

Abdominal pregnancy has been defined as an embryonic implantation in the peritoneal cavity, exclusive of tubal, ovarian, or intraligamentary implantations [1]. It accounts for 0.6 to 4% of all ectopic pregnancies and 1 in 3372 to 1 in 21,439 live births [2]. Abdominal gestations are usually secondary to early tubal rupture with subsequent implantation onto the peritoneal surfaces [3].

High index of suspicion is important to diagnose abdominal pregnancy. In contrast to tubal ectopic pregnancies, abdominal pregnancies may go undetected until an advanced gestational age. As a result, it is associated with a high rate of maternal complications [4]. The maternal mortality rate can be as high as 20%. This is primarily because of the risk of massive hemorrhage from partial or total placental separation. The placenta can be attached to the uterine wall, bowel, mesentery, liver, spleen, bladder and ligaments [5].

It is noted that the classical signs and symptoms of ectopic pregnancy are usually present in those cases generally regarded as secondary abdominal ectopic pregnancies, but the symptoms are quite variable in regard to those cases recognized as primary [6]. The clinical presentation in our case was vaginal bleeding of three days duration and it was initially mis-diagnosed as a missed second trimester abortion.

**Fig. 1.**
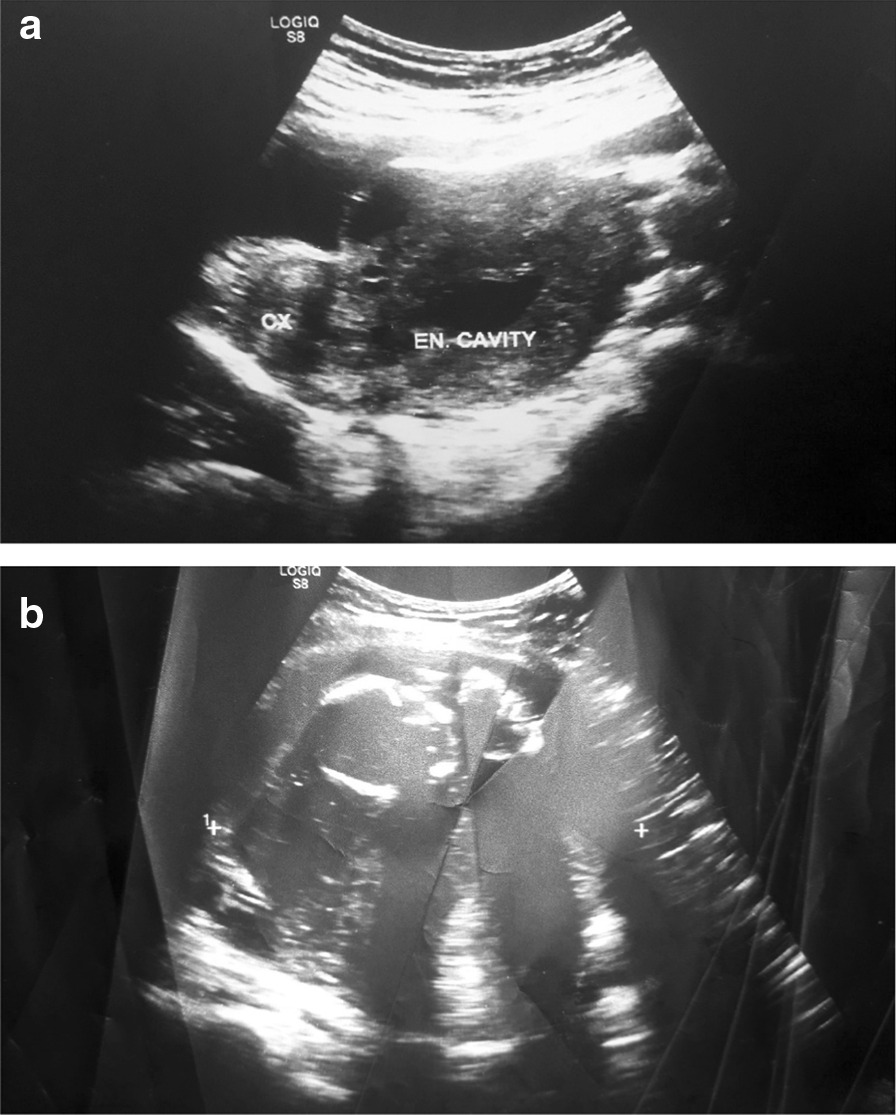
Pelvic ultrasound findings. **a** Demonstrates an empty uterus and **b** depicts a well-formed embryo in close proximity to the anterior abdomen al wall with the placenta positioned in the posterior abdominal cavity

**Fig. 2.**
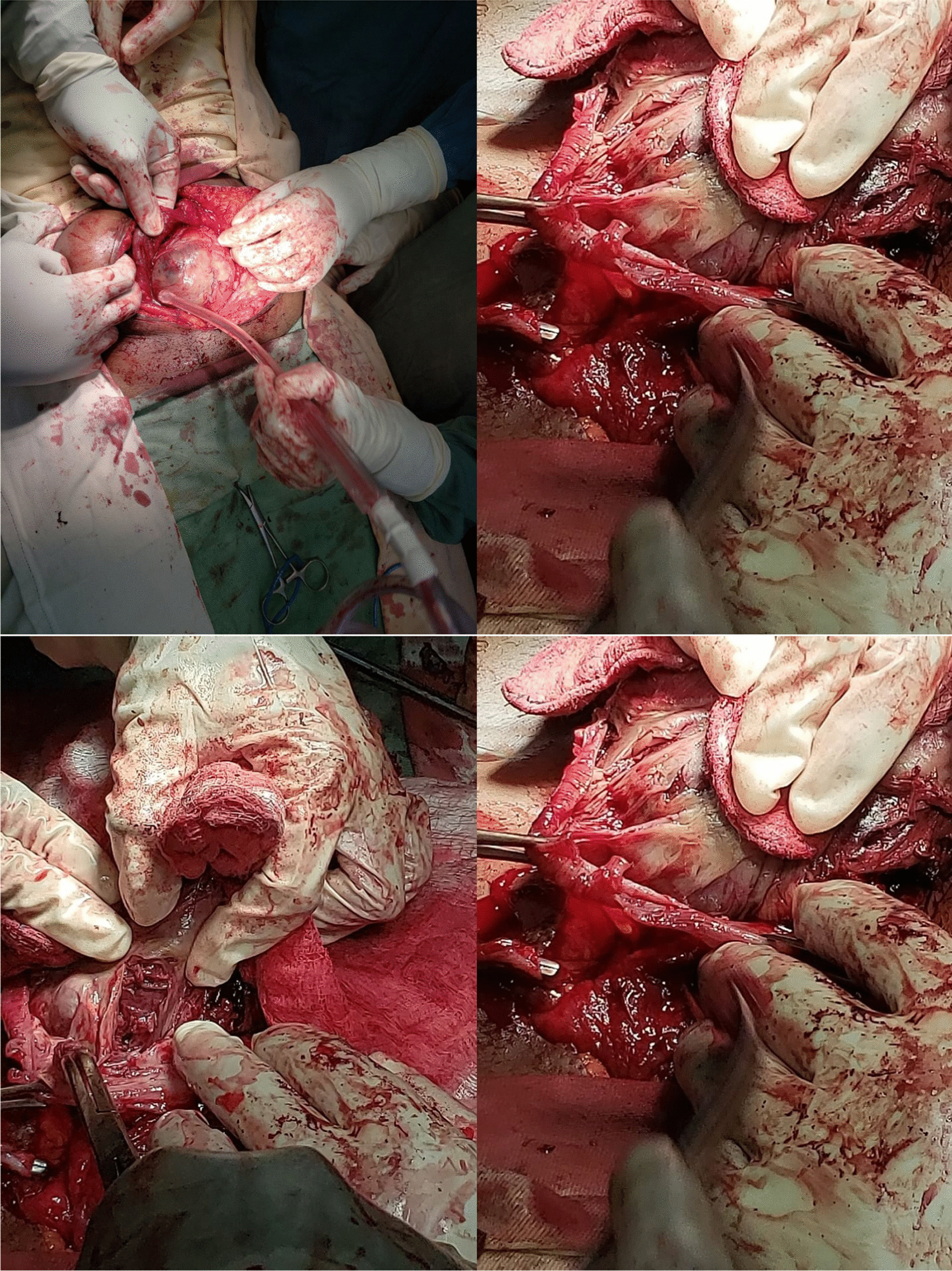

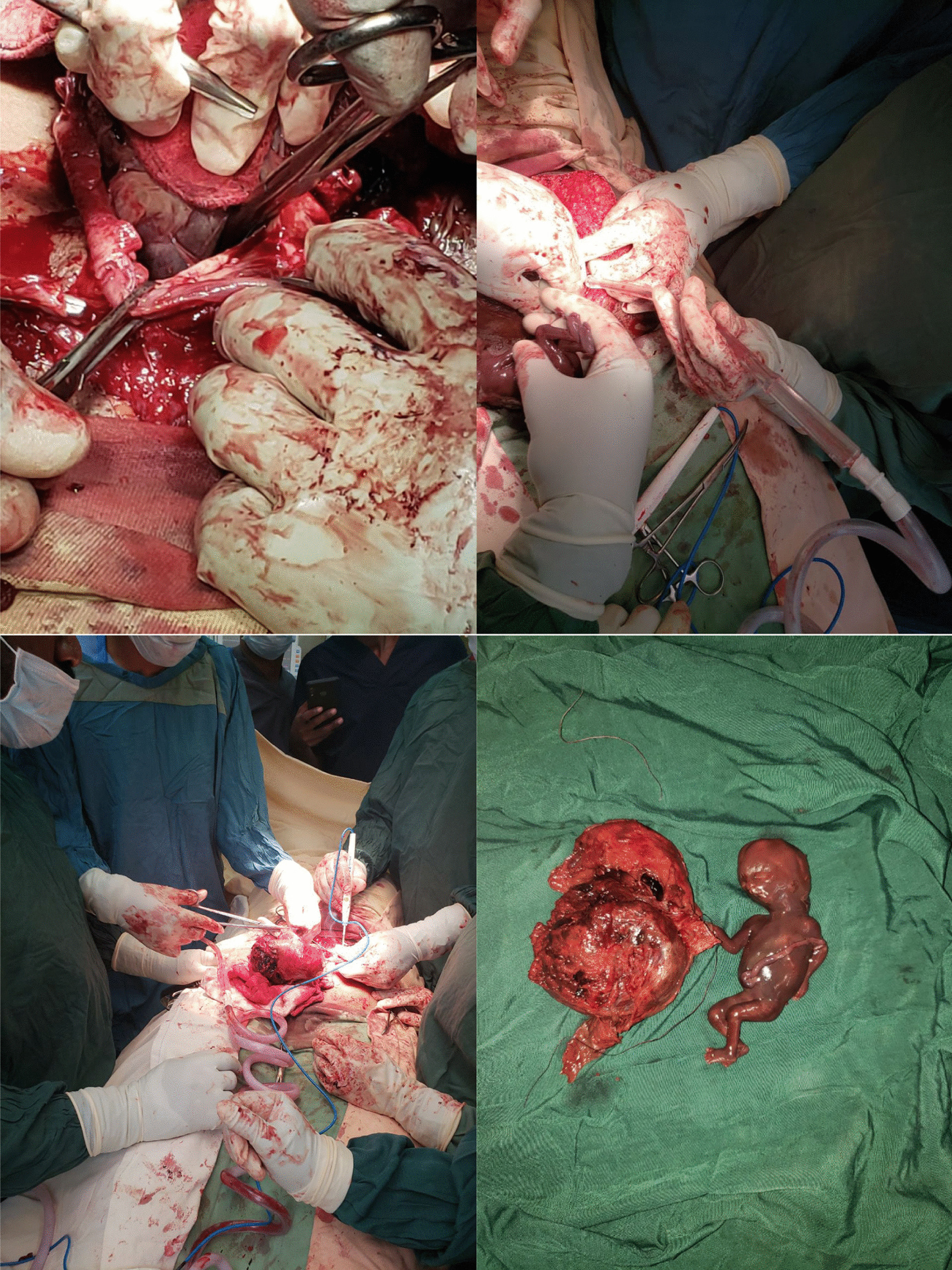
The whole surgery captured in real time photos

With ultrasound examination, an empty uterus coupled with the presence of a gestational sac or mass separated from the uterus, adnexa and ovaries should always raise suspicion of an abdominal pregnancy. A mass seen in the abdomen away from the pelvis, especially with features of pregnancy (gestational and yolk sac, fetal heart beat) is diagnostic but it is not usually possible to differentiate a pelvic mass from the adnexa with ultrasound [7]. In our case, there was a clearly visible well-formed demised fetus out of the uterine cavity (Figs. 1, 2).

The mainstay of management for abdominal pregnancy is surgery. Removal of the ectopic pregnancy mass could cause intractable hemorrhage and/or organ injury because of deep trophoblastic invasion into the surrounding tissue [8]. Management of the placenta presents the greatest problem [9]. Immediate operation should be done once a diagnosis of abdominal pregnancy is entertained and an adequate supply of compatible Rh suitable blood has to be prepared [10].

The surgeon rarely encounters difficulty in opening the sac and removing the fetus, but management of the sac, the adhesions, and more especially of the placenta, and control of hemorrhage present surgical problems of the first magnitude. The ideal procedure, of course, is to remove the sac in toto: fetus, membranes and placenta. The surgeon should be prepared to deal with profuse bleeding because as soon as he or she begins to strip off the placenta he may encounter violent hemorrhage which cannot be controlled by clamps, stitches or packing. If the placenta is adherent to the intestines, liver or spleen, separation is likely to produce uncontrollable bleeding; the placenta should be left *in situ* and the abdomen closed without drainage [11].

In our case, there was no difficulty in removing the fetus and placenta but there was inadvertent injury to the sigmoid colon while an attempt was made to release the dense adhesion between the ectopic mass and the large bowel.

## Conclusion

Most of the recommendations for the management of abdominal ectopic pregnancy focus on the precautions that has to be taken in making a sound surgical examination to examine the extent of placental attachment to the surrounding tissue, after entry to the abdominal cavity. This aims to avoid massive hemorrhage and organ injury by making a good clinical judgment whether to leave the placenta *in situ* or remove it. What is equally important and can result in potential organ injury if the necessary precautions are not taken, like the sigmoid colon injury which occurred in our case, is safe entry to the abdomen.

We recommend that the possibility of bowel injury during entry to the abdominal cavity at laparotomy should always be considered and an experienced general surgeon should always be in attendance before opening the abdomen, to prevent it from happening. We also recommend that a differential diagnosis of abdominal ectopic pregnancy should be considered and a repeat ultrasound examination should be done in any case of second trimester failed medication abortion.

## Availability of supporting data

All supporting documents are submitted along with the case report.
